# Comparative Evaluation of Three Endodontic File Systems for Pericervical Dentin Preservation and Canal Transportation Using Cone-Beam Computed Tomography: An In Vivo Study

**DOI:** 10.7759/cureus.116400

**Published:** 2026-09-17

**Authors:** Nakul Suresh, Rishi Manan, Saumya Vats, Kirti Rathee, Bharat Chauhan, Ajay B Gutti

**Affiliations:** 1 Department of Conservative Dentistry and Endodontics, Institute of Dental Studies and Technologies, Modinagar, IND; 2 Department of Conservative Dentistry and Endodontics, Malla Reddy Institute of Dental Sciences, Hyderabad, IND

**Keywords:** cone-beam computed tomography, endodontics, nickel-titanium, root canal preparation, tooth root

## Abstract

Introduction: The preservation of pericervical dentin (PCD) and maintenance of the original root canal anatomy are fundamental objectives of contemporary minimally invasive endodontics. Advances in nickel-titanium (NiTi) rotary instrumentation have led to the development of file systems designed to improve shaping efficiency while minimizing dentin removal and canal transportation. Therefore, our aim was to compare the effects of GenEndo (Coltene Holding AG, Altstätten, Switzerland), ProTaper Ultimate (Dentsply Sirona, Ballaigues, Switzerland), and TruNatomy (Dentsply Sirona, Ballaigues, Switzerland) rotary file systems on PCD preservation and canal transportation using cone-beam computed tomography (CBCT).

Materials and methods: This prospective in vivo comparative clinical study included 30 mandibular first molars requiring primary root canal treatment. The teeth were categorized into three groups (*n* = 10 each) according to the rotary file system used: GenEndo, ProTaper Ultimate, and TruNatomy. Preoperative and postoperative CBCT scans were obtained to evaluate PCD thickness and canal transportation. PCD was measured 4 mm apical to the cemento-enamel junction at the buccal, lingual, mesial, and distal aspects. Canal transportation was calculated by comparing pre- and post-instrumentation CBCT measurements. Data were analyzed using one-way analysis of variance followed by Tukey's post hoc test, with statistical significance set at p < 0.05.

Results: Significant intergroup differences were observed in PCD preservation at all evaluated surfaces (p < 0.05). TruNatomy demonstrated the greatest preservation of PCD, followed by GenEndo, whereas ProTaper Ultimate exhibited the greatest dentin removal. Canal transportation also differed significantly among the groups (*P* = 0.009). TruNatomy produced the lowest mean canal transportation (0.0160 ± 0.0158 mm), significantly lower than both GenEndo (*P* = 0.043) and ProTaper Ultimate (*P* = 0.007). No significant difference in canal transportation was observed between GenEndo and ProTaper Ultimate (*P* = 0.951).

Conclusions: Within the limitations of this prospective in vivo clinical study, TruNatomy demonstrated superior preservation of PCD and significantly reduced canal transportation compared with GenEndo and ProTaper Ultimate. These findings support the use of heat-treated NiTi rotary systems to maintain root canal anatomy while preserving critical dentin during endodontic treatment.

## Introduction

Successful endodontic treatment depends on effective cleaning and shaping of the root canal system while preserving its original anatomy. Biomechanical preparation is a critical stage of root canal therapy because it facilitates irrigation, disinfection, and obturation [1]. However, excessive dentin removal during instrumentation may weaken the tooth structure and increase the risk of procedural errors, such as canal transportation, ledge formation, strip perforation, and vertical root fracture [2]. Therefore, contemporary endodontics emphasizes minimally invasive approaches that achieve adequate canal debridement while conserving sound dentin [3].

Pericervical dentin (PCD), extending approximately 4 mm above and below the alveolar crest, is considered the structural foundation of an endodontically treated tooth [4]. This region plays a crucial role in transmitting occlusal forces and maintaining fracture resistance. Preservation of the PCD has been associated with improved long-term survival of root canal-treated teeth, whereas unnecessary removal of dentin in this area may compromise tooth strength [5]. Similarly, canal transportation, defined as the deviation of the prepared canal from its original path due to unequal dentin removal, adversely affects cleaning efficiency, apical sealing, and overall treatment prognosis [6]. Consequently, both PCD preservation and minimal canal transportation have become important parameters for evaluating the shaping abilities of contemporary nickel-titanium (NiTi) rotary instruments.

Advances in NiTi metallurgy and instrument design have led to the development of newer rotary systems that aim to improve flexibility, maintain canal curvature, and preserve dentin [7]. TruNatomy (Dentsply Sirona, Ballaigues, Switzerland) incorporates a heat-treated NiTi alloy with a slim core and regressive taper to promote conservative canal shaping [8]. ProTaper Ultimate (Dentsply Sirona, Ballaigues, Switzerland) represents the latest evolution of the ProTaper series, with advanced heat-treated alloys designed to enhance flexibility and fatigue resistance [9]. GenEndo (Coltene Holding AG, Altstätten, Switzerland) is a recently introduced rotary system utilizing T-wire technology to improve flexibility while reducing unnecessary dentin removal [10]. Despite these innovations, limited clinical evidence is available comparing the performance of these contemporary file systems under in vivo conditions. Cone-beam computed tomography (CBCT), with its high-resolution three-dimensional imaging capability, enables accurate assessment of dentin thickness and canal transportation before and after instrumentation, making it a reliable method for such evaluations [11].

The present study aimed to evaluate and compare the effects of GenEndo, TruNatomy, and ProTaper Ultimate rotary file systems on PCD preservation and canal transportation using CBCT in vivo. The specific objectives were to: (1) compare the amount of PCD preserved following instrumentation with GenEndo, TruNatomy, and ProTaper Ultimate rotary file systems; (2) evaluate and compare the degree of canal transportation produced by the three rotary systems using CBCT; and (3) compare the overall shaping performance of the three rotary file systems with respect to PCD preservation and canal transportation.

## Materials and methods

Study design

This prospective in vivo comparative clinical study was conducted at the Department of Conservative Dentistry and Endodontics, IDST Dental College and Hospital, Modinagar, Uttar Pradesh, India, between May 2023 and October 2023. Because the study evaluated routinely used rotary instrumentation systems during conventional endodontic treatment without a randomized controlled trial design, it was conducted as a prospective comparative clinical investigation. Ethical approval for the study was obtained from the Institutional Ethics Committee (IDST/IEC/2022-25/06, dated April 25, 2023) before patient recruitment, and written informed consent was obtained from all participants before inclusion in the study. All procedures were performed in accordance with the ethical principles outlined in the Declaration of Helsinki.

Sample size estimation

The sample size was estimated using G*Power software (version 3.1.9.7; Heinrich Heine University, Düsseldorf, Germany) by performing an a priori power analysis for a one-way analysis of variance (ANOVA). Based on a previous study, an effect size (*f*) of 0.50, statistical power of 80% (1 − *β* = 0.80), and two-sided alpha level of 0.05 were considered [12]. The minimum required sample size was calculated as 30 teeth, with 10 specimens in each study group.

Following confirmation of eligibility, the included teeth were randomly allocated in a 1:1:1 ratio to the three instrumentation groups (*n* = 10 per group) using a computer-generated randomization sequence. The allocation sequence was generated before commencement of instrumentation, and each eligible tooth was assigned to one of the three rotary file systems according to the predetermined sequence.

Eligibility criteria

Patients aged between 16 and 60 years requiring primary root canal treatment in mandibular first molars diagnosed with irreversible pulpitis, apical periodontitis, or requiring intentional root canal therapy were screened for eligibility. Only teeth with fully developed roots, clinically restorable crowns, and suitable root canal morphologies were included. Patients with teeth that had undergone previous endodontic treatment, gross coronal destruction, open apices, root fractures, root resorption, acute periapical abscesses, developmental anomalies, calcified canals, or abnormal root canal morphology were excluded. Patients with systemic conditions contraindicating routine endodontic treatment or those who were unwilling to participate were also excluded. Only one mandibular first molar was included from each patient to ensure statistical independence of the observations.

Examiner calibration, standardization, and reliability

Before the commencement of the study, the principal investigator underwent calibration under the supervision of a senior endodontist experienced in the CBCT-based assessment of root canal morphology. Ten CBCT scans that were not included in the study sample were used for examiner training and calibration. Standard operating procedures were established for patient positioning, CBCT acquisition, biomechanical preparation, and image analysis to ensure methodological consistency throughout the study. PCD thickness and canal transportation measurements were repeated after a two-week interval under identical viewing conditions by the same examiner. Intra-examiner reliability was assessed using the intraclass correlation coefficient (ICC), with an ICC value of ≥0.80 considered indicative of excellent agreement. All CBCT measurements were performed by a single calibrated examiner to minimize inter-examiner variability.

Clinical procedure

Following clinical examination and confirmation of eligibility, preoperative intraoral periapical radiographs were obtained to evaluate the tooth and periapical status. Because of the inherent limitations of two-dimensional imaging, CBCT imaging was subsequently performed to obtain a three-dimensional assessment of the canal anatomy and baseline PCD thickness before instrumentation. Local anesthesia was administered using 2% lignocaine with 1:100,000 epinephrine (Indoco Remedies Ltd., Mumbai, Maharashtra, India). The tooth was isolated using a rubber dam (Coltene Holding AG, Altstätten, Switzerland). All clinical procedures were performed under 3.5× magnification loupes (Q-Optics Inc., Duncanville, TX) by the same experienced operator to minimize variability. Conventional access cavity preparation was performed using Endo Access burs (MANI Inc., Tochigi, Japan). Canal patency was established using a #10 stainless steel K-file (Dentsply Sirona, Ballaigues, Switzerland). The working length was determined using an electronic apex locator and confirmed radiographically before the commencement of biomechanical preparation.

Thirty mandibular first molars were categorized into three study groups according to the rotary instrumentation system used for canal preparation: Group I, GenEndo rotary file system; Group II, ProTaper Ultimate rotary file system; and Group III, TruNatomy rotary file system. Biomechanical preparation in each group was performed according to the respective manufacturers’ recommended sequence and operating parameters. Throughout instrumentation, the canals were lubricated with 17% ethylenediaminetetraacetic acid. (EDTA) gel, and irrigation was carried out using 3% sodium hypochlorite and normal saline delivered through a 30-gauge side-vented irrigation needle (Ultradent Products Inc., South Jordan, UT). The irrigants were activated using an ultrasonic activation device (Waldent Innovations Pvt. Ltd., New Delhi, India), followed by sonic activation using the Super Endo Activator (Super Endo, India). A final flush with sterile normal saline was performed before the completion of canal preparation.

CBCT assessment

Following the completion of biomechanical preparation, postoperative CBCT scans were acquired using the CS 8100 3D Cone Beam Imaging System (Carestream Dental LLC, Atlanta, GA) with the same acquisition parameters as the preoperative scans to ensure standardization. All scans were obtained using a 5 × 5 cm field of view (FOV), 75-µm isotropic voxel size, 90 kVp tube voltage, 5 mA tube current, and an exposure time of approximately 10.8 s. The images were reconstructed and analyzed using the manufacturer's proprietary software under standardized viewing conditions. Preoperative and postoperative CBCT images were evaluated by the same calibrated examiner in a dimly lit room using a high-resolution monitor. To minimize measurement bias, the examiner was blinded to the previous measurements during the image evaluation.

Axial sections of 1-mm thickness were used to assess PCD thickness and canal transportation. All measurements were recorded twice at two-week intervals, and the mean values were used for statistical analysis. The PCD thickness was measured at a level 4 mm apical to the cementoenamel junction on the buccal, lingual, mesial, and distal aspects using 1-mm axial sections. The minimum distance between the prepared canal wall and the corresponding external root surface was recorded. All PCD thickness and canal transportation measurements were performed on the mesial root at the level of the mesiobuccal canal. PCD thickness was measured at the buccal, lingual, mesial, and distal aspects of the mesial root. Canal transportation was calculated at the same cross-sectional level using the mesial and distal external root surfaces as reference points.

Canal transportation was determined by comparing preoperative and postoperative CBCT images using the formula described by Gambill et al. [13]:

\[ \text{Canal Transportation} = (A_1 - A_2) - (B_1 - B_2) \]

where A1 represents the shortest distance from the mesial external root surface to the mesial canal wall before instrumentation, A2 represents the corresponding distance after instrumentation, B1 represents the shortest distance from the distal external root surface to the distal canal wall before instrumentation, and B2 represents the corresponding distance after instrumentation (Figure 1). A value of zero indicates perfect centering, whereas positive and negative values represent transportation in opposite directions.

**Figure 1:**
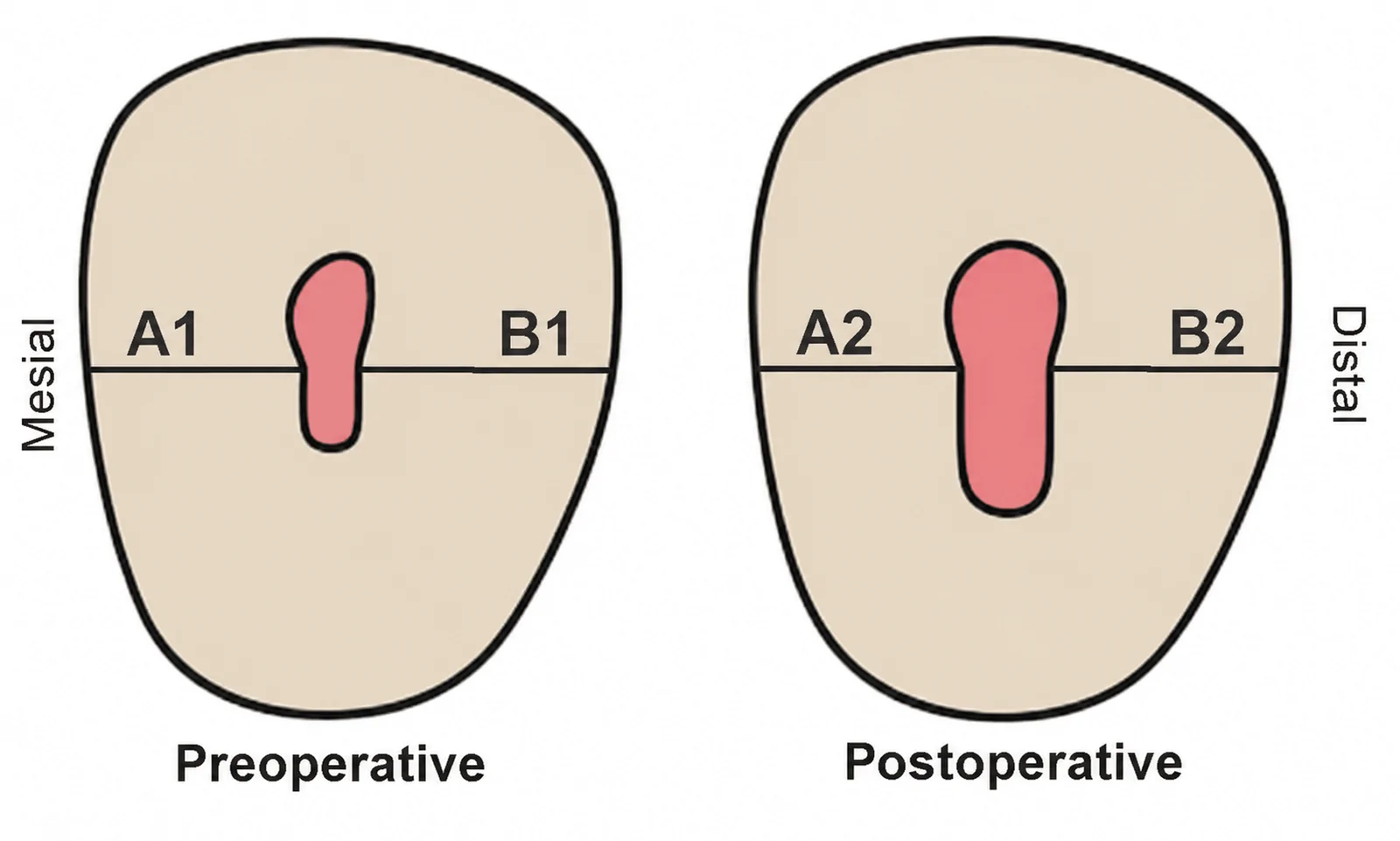
Cross-sectional schematic of the root canal before and after rotary instrumentation. The preoperative panel shows the original canal morphology, while the postoperative panel shows an enlarged, uniformly prepared canal. Mesial and distal orientations are indicated for comparison of canal enlargement and dentin thickness. A1 represents the shortest distance from the mesial external root surface to the mesial canal wall before instrumentation; A2 represents the corresponding distance after instrumentation; B1 represents the shortest distance from the distal external root surface to the distal canal wall before instrumentation; and B2 represents the corresponding distance after instrumentation. The figure was created using Canva (Canva Pty Ltd., Sydney, New South Wales, Australia), and no artificial intelligence (AI) features or AI-assisted tools available within Canva were used during its preparation.

Outcome measures

The primary outcome measure was the amount of PCD preserved after biomechanical preparation. The secondary outcome measure was the degree of canal transportation produced by each rotary instrumentation system.

Statistical analysis

Statistical analyses were performed using IBM SPSS Statistics for Windows, version 26.0 (IBM Corp., Armonk, NY). Data are expressed as mean ± standard deviation (SD). The normality of the data distribution was assessed using the Shapiro-Wilk test. As the data demonstrated a normal distribution, intergroup comparisons were performed using one-way ANOVA for continuous variables, and the chi-square test was used for categorical variables such as sex. When statistically significant differences were identified, Tukey's post hoc multiple comparison test was applied for pairwise comparisons. Effect sizes (η² for ANOVA and Cohen's *d* for pairwise comparisons) were calculated to determine the magnitude of the differences between the study groups. Statistical significance was set at *P* < 0.05.

## Results

Thirty mandibular first molars were included in the study, with 10 teeth evaluated in each of the three study groups. The demographic analysis confirmed baseline comparability across the three study groups. One-way ANOVA revealed no significant age differences (*F* = 0.34, *P* = 0.714), while the chi-square test confirmed a perfectly balanced sex distribution (χ² = 0.00, *P* = 1.000). These findings establish group homogeneity, ensuring that subsequent outcome comparisons are not confounded by demographic imbalances (Table 1).

**Table 1:** Demographic details of study participants. Data are expressed as mean ± SD or *n* (%). Intergroup comparisons were performed using one-way analysis of variance (ANOVA) for age and the chi-square test for sex. SD, standard deviation

Parameters		GenEndo	ProTaper Ultimate	TruNatomy	Test value	*P*-value
Age, mean ± SD	Years	26.50 ± 4.35	25.34 ± 5.65	27.12 ± 4.60	*F* = 0.34	0.714
Sex, *n* (%)	Male	5 (50)	5 (50)	5 (50)	χ² = 0.00	1.000
	Female	5 (50)	5 (50)	5 (50)		

Intergroup comparisons of PCD preservation and canal transportation were performed after biomechanical preparation using the three rotary file systems. Statistically significant differences were observed among the three groups for PCD preservation at the buccal, lingual, mesial, and distal aspects (*P* < 0.05). The TruNatomy group demonstrated the lowest mean dentin removal at all evaluated surfaces, indicating superior preservation of the peri-cervical dentin. ProTaper Ultimate exhibited the greatest dentin removal, whereas GenEndo showed intermediate values between the two systems (Table 2).

**Table 2:** Comparison of pericervical dentin preservation (mm) among the three rotary file systems. Data are presented as mean ± standard deviation (SD) with 95% confidence intervals (CI). Intergroup comparisons were performed using one-way analysis of variance (ANOVA). Effect size is expressed as eta squared (η²). ^*^P < 0.05 was considered statistically significant. PCD-B, buccal pericervical dentin; PCD-L, lingual pericervical dentin; PCD-M, mesial pericervical dentin; PCD-D, distal pericervical dentin

Tooth aspect	Groups	N	Mean ± SD (mm)	95% CI	*F*-statistics	*P*-value	Effect size (η²)
PCD-B	GenEndo	10	0.2420 ± 0.0464	0.2088-0.2752	10.428	0.0004*	0.436
	ProTaper Ultimate	10	0.4760 ± 0.3016	0.2603-0.6917			
	TruNatomy	10	0.1200 ± 0.0323	0.0969-0.1431			
PCD-L	GenEndo	10	0.3740 ± 0.1833	0.2429-0.5051	15.230	< 0.0001*	0.530
	ProTaper Ultimate	10	0.3940 ± 0.1456	0.2899-0.4981			
	TruNatomy	10	0.0930 ± 0.0316	0.0704-0.1156			
PCD-M	GenEndo	10	0.2770 ± 0.2182	0.1209-0.4331	4.708	0.0175*	0.259
	ProTaper Ultimate	10	0.4570 ± 0.3377	0.2154-0.6986			
	TruNatomy	10	0.1350 ± 0.0652	0.0884-0.1816			
PCD-D	GenEndo	10	0.2960 ± 0.1840	0.1644-0.4276	6.648	0.0044*	0.330
	ProTaper Ultimate	10	0.4960 ± 0.3455	0.2489-0.7431			
	TruNatomy	10	0.1200 ± 0.0806	0.0624-0.1776			

Pairwise comparisons using Tukey's post hoc test revealed that TruNatomy preserved significantly more PCD than ProTaper Ultimate in all the evaluated aspects. At the buccal and lingual surfaces, TruNatomy also demonstrated significantly greater dentin preservation than GenEndo, whereas no significant differences were observed between GenEndo and TruNatomy at the mesial and distal surfaces (Table 3).

**Table 3:** Pairwise comparison of pericervical dentin preservation (mm) among the three rotary file systems using Tukey's post hoc test. Pairwise comparisons were performed using Tukey’s honestly significant difference (HSD) post hoc test following one-way ANOVA. ^*^P < 0.05 was considered statistically significant. PCD-B, buccal pericervical dentin; PCD-L, lingual pericervical dentin; PCD-M, mesial pericervical dentin; PCD-D, distal pericervical dentin; ANOVA, analysis of variance

Tooth aspect	GenEndo vs. ProTaper Ultimate		GenEndo vs. TruNatomy		ProTaper Ultimate vs. TruNatomy	
	t-statistics	*P*-value	t-statistics	*P*-value	t-statistics	*P*-value
PCD-B	2.953	0.035*	1.540	0.420	4.493	<0.001*
PCD-L	0.328	0.945	4.607	<0.001*	4.935	<0.001*
PCD-M	1.711	0.280	1.350	0.520	3.062	0.027*
PCD-D	1.938	0.160	1.705	0.280	3.644	0.008*

A statistically significant difference was observed between the three instrumentation systems (p = 0.009). TruNatomy produced the lowest mean canal transportation (0.0160 ± 0.0158 mm), followed by GenEndo (0.1220 ± 0.1116 mm), whereas ProTaper Ultimate demonstrated the highest transportation (0.1440 ± 0.1108 mm), as shown in Table 4.

**Table 4:** Comparison of canal transportation (mm) among the three rotary file systems. Data are presented as mean ± standard deviation (SD). Intergroup comparisons were performed using one-way analysis of variance (ANOVA). Effect size is expressed as eta squared (η²). *P < 0.05 was considered statistically significant.

Groups	N	Mean ± SD	*F*-statistics	*P*-value	Effect size (η²)
GenEndo	10	0.1220 ± 0.1116	5.626	0.009*	0.294
ProTaper Ultimate	10	0.1440 ± 0.1108			
TruNatomy	10	0.0160 ± 0.0158			

According to Tukey’s post hoc analysis, canal transportation was significantly lower with TruNatomy than with both GenEndo (*P* = 0.043) and ProTaper Ultimate (*P* = 0.007). However, no statistically significant difference was observed between the GenEndo and ProTaper Ultimate groups (*P* = 0.951), as shown in Table 5.

**Table 5:** Pairwise comparison of canal transportation (mm) among the three rotary file systems using Tukey's post hoc test. Pairwise comparisons were performed using Tukey’s honestly significant difference (HSD) post hoc test following one-way ANOVA. ^*^P < 0.05 was considered statistically significant. ANOVA, analysis of variance

Tooth aspect/Pairwise comparison	Mean difference (mm)	*t* statistics	*P*-value (Tukey adjusted)	Effect size (Cohen's *d*)
GenEndo vs. ProTaper Ultimate	-0.0220	-0.539	0.951	-0.241
GenEndo vs. TruNatomy	0.1060	2.597	0.043*	1.162
ProTaper Ultimate vs. TruNatomy	0.1280	3.137	0.007*	1.403

## Discussion

This prospective in vivo clinical study compared the shaping performance of three contemporary NiTi rotary file systems, GenEndo, ProTaper Ultimate, and TruNatomy, with respect to PCD preservation and canal transportation using CBCT. The findings demonstrated significant differences among the three systems, with TruNatomy showing the greatest preservation of PCD and the least canal transportation, followed by GenEndo. ProTaper Ultimate exhibited comparatively greater dentin removal and canal transportation than the other systems.

The preservation of the PCD has become a major objective of minimally invasive endodontics because this region plays a fundamental role in distributing occlusal stresses and maintaining the structural integrity of endodontically treated teeth [5]. Excessive dentin removal in this critical zone may increase susceptibility to vertical root fractures and compromise long-term tooth survival [14]. Therefore, instrumentation systems capable of achieving effective canal preparation while conserving dentin are advantageous.

The superior performance of TruNatomy observed in this study may be primarily attributed to its unique design characteristics. The system is manufactured from a proprietary heat-treated NiTi alloy and incorporates a slim 0.8-mm wire, regressive taper, and off-centered parallelogram cross-section, which collectively improve flexibility while reducing unnecessary engagement with the canal walls [15]. These design characteristics enable the instrument to follow the original canal anatomy with minimal removal of sound dentin, particularly within the pericervical region [8,15].

The comparatively greater dentin removal observed with ProTaper Ultimate in the present study may be attributed to its progressively variable taper, which facilitates efficient canal shaping but may result in increased coronal dentin removal compared with conservative systems, such as TruNatomy. Similar observations have been reported for the ProTaper series in a micro-computed tomography study [16]. GenEndo demonstrated intermediate performance, likely because its T-wire metallurgy and conservative 4% taper provide improved flexibility while maintaining adequate cutting efficiency [10]. The relatively high variability observed for some PCD measurements, particularly in the ProTaper Ultimate group, should also be considered when interpreting the findings. Although the sample size was determined a priori, the small number of teeth per group may limit the precision of estimates in the presence of substantial inter-tooth variability. Therefore, the findings should be interpreted cautiously and confirmed in studies with larger sample sizes.

The findings of the present study are consistent with those reported by Silva et al. [16], who demonstrated that TruNatomy preserved significantly more coronal periradicular dentin than the ProTaper Gold system while maintaining comparable shaping abilities and minimal canal transportation in mandibular molars. Furthermore, Tabassum et al. [17] highlighted that the design philosophy of TruNatomy emphasizes minimally invasive endodontics by preserving the radicular dentin while providing adequate canal enlargement for effective cleaning and irrigation. Collectively, these observations support the present findings and reinforce the clinical advantages of conservative rotary instrumentation. Although the present study demonstrated significantly lower canal transportation with TruNatomy, Kabil et al. [18] reported no significant differences in canal transportation among TruNatomy, ProTaper Next, and XP-Endo Shaper. This discrepancy may be attributed to differences in the instrumentation systems evaluated, canal anatomy, sample selection, and study methodology.

The GenEndo rotary system demonstrated better preservation of PCD than ProTaper Ultimate, although it did not perform as well as the TruNatomy rotary system. This observation may be explained by its T-wire technology and smaller constant taper, which enhance instrument flexibility and reduce aggressive dentin cutting [10,17]. Karkehabadi et al. [19] reported that GenEndo preserved greater remaining dentin thickness and produced less canal transportation than ProTaper Universal, supporting the present results. Although clinical evidence regarding GenEndo remains limited, the available literature suggests that its conservative design contributes to favorable shaping outcomes.

In contrast to the present findings, Langaliya et al. [20] evaluated the XP-Endo Shaper, Self-Adjusting File (SAF), HyFlex EDM, ProTaper Next, WaveOne Gold, and hand K-files using CBCT and reported that the XP-Endo Shaper demonstrated the least canal transportation and superior centering ability compared with the other evaluated instrumentation systems. The discrepancy between their findings and those of the present study is likely attributable to differences in the rotary systems investigated, canal morphology, study design, instrumentation protocols, and distinct metallurgical and geometric characteristics of the instruments. Unlike the present study, which evaluated TruNatomy, ProTaper Ultimate, and GenEndo, their investigation did not include these novel rotary systems.

Canal transportation remains one of the most important indicators of shaping quality because deviations from the original canal path may compromise irrigation effectiveness, obturation quality, and long-term treatment success. In the present study, TruNatomy produced significantly lower canal transportation than GenEndo and ProTaper Ultimate. This finding is most likely related to its exceptional flexibility, reduced core diameter, and non-cutting tip, which allow the instrument to negotiate the canal curvature while maintaining the original canal trajectory [15,17].

These findings are consistent with those of Silva et al. [16], who demonstrated that TruNatomy preserved periradicular dentin while maintaining effective canal shaping with minimal transportation in mandibular molars. Likewise, Zargar et al. [21] demonstrated that TruNatomy produces minimal canal transportation in curved canals because of its regressive taper and improved flexibility. Similar findings were reported by Kabil et al. [18], further supporting the ability of TruNatomy to preserve the original canal anatomy during instrumentation. Collectively, these studies provide strong evidence to support the shaping efficiency observed in the present investigation.

An important strength of the present study was its in vivo design. Most previous investigations comparing contemporary rotary systems have been performed on extracted teeth under laboratory conditions. By evaluating teeth during routine clinical treatment, the present study provides findings that are more representative of actual clinical practices. Furthermore, the use of CBCT enabled a three-dimensional assessment of dentin thickness and canal transportation with greater accuracy than conventional two-dimensional radiography, thereby enhancing the reliability of the measurements.

Although preservation of PCD is an important objective of minimally invasive endodontics, it must be balanced against adequate canal cleaning and disinfection. Excessive conservation of dentin should not compromise effective debridement or irrigation. The present study did not evaluate untouched canal walls, microbial reduction, or cleaning efficacy; therefore, the greater PCD preservation observed with TruNatomy cannot be interpreted as evidence of overall clinical superiority. Rather, the findings indicate an advantage in terms of structural preservation and reduced canal transportation, while the balance between conservative shaping and cleaning efficacy requires further investigation.

The clinical significance of the observed differences in canal transportation should also be interpreted cautiously. Although statistically significant differences were observed among the instrumentation systems, the present study did not evaluate whether these differences translate into clinically meaningful improvements in treatment outcomes, fracture resistance, or long-term tooth survival. Furthermore, the small sample size, absence of quantitative baseline canal-curvature assessment, and limitations associated with the 75-µm CBCT voxel size may have influenced the observed results. Therefore, the findings should be considered specific to the evaluated mandibular first molars and study conditions and should not be interpreted as evidence of broad clinical superiority of any particular instrumentation system.

Clinical implications

The findings of the present study have important clinical implications for contemporary endodontics. Preservation of the PCD is directly associated with improved fracture resistance and long-term survival of endodontically treated teeth. Therefore, rotary instrumentation systems that minimize unnecessary dentin removal while maintaining the original canal anatomy should be preferred, particularly for teeth with thin root walls or complex canal curvatures. Based on the present findings, TruNatomy appears to be a suitable option for minimally invasive endodontic treatment because it provides effective canal shaping while preserving critical dentin and minimizing canal transportation. GenEndo also demonstrated satisfactory shaping performance and may represent a conservative alternative, whereas clinicians should exercise greater caution when using larger taper systems in anatomically challenging canals.

Limitations

The present study had certain limitations. The relatively small sample size may limit the generalizability of the findings. Furthermore, only mandibular first molars were included; therefore, the results may not be directly applicable to other tooth types with different anatomical characteristics. The study focused on PCD preservation and canal transportation, while other clinically relevant outcomes, including shaping time, debris extrusion, postoperative pain, cyclic fatigue resistance, and long-term treatment outcomes, were not evaluated. Preoperative root canal curvature was also not quantitatively assessed using the Schneider angle or radius of curvature. Because canal curvature may influence canal transportation and shaping behavior, the absence of standardized baseline curvature measurements may have introduced anatomical variability among the study groups. In addition, the use of different manufacturer-recommended speed, torque, and instrumentation parameters among the three file systems may have influenced the shaping outcomes; therefore, the observed differences cannot be attributed solely to file design and metallurgy. Although CBCT provides three-dimensional assessment, its spatial resolution is lower than that of micro-computed tomography. The relatively small magnitude of some measured changes should also be interpreted in relation to the spatial resolution of the CBCT system used in this study. Although a 75-µm isotropic voxel size was used, measurements below or close to the voxel dimension may be susceptible to partial-volume effects and measurement uncertainty. Therefore, very small differences in canal transportation should be interpreted cautiously and should not be considered equivalent to changes that can be resolved by micro-computed tomography. Future studies using higher-resolution micro-computed tomography or validated measurement-error analyses are recommended to confirm small dimensional changes. Future multicenter studies with larger sample sizes, standardized assessment of canal curvature and instrumentation parameters, longer follow-up periods, and evaluation of additional clinical outcomes are warranted to validate these findings.

## Conclusions

Within the limitations of this prospective in vivo clinical study, TruNatomy demonstrated greater preservation of PCD and less canal transportation than the other evaluated rotary systems in mandibular first molars. GenEndo showed intermediate performance, whereas ProTaper Ultimate demonstrated comparatively greater dentin removal and canal transportation. These findings suggest potential advantages of TruNatomy for preserving tooth structure and maintaining canal anatomy under the conditions evaluated; however, further studies involving larger samples and diverse tooth types are required to determine the broader clinical relevance of these findings.
